# Neuron-Glia Interactions in Neural Plasticity: Contributions of Neural Extracellular Matrix and Perineuronal Nets

**DOI:** 10.1155/2016/5214961

**Published:** 2016-01-05

**Authors:** Egor Dzyubenko, Christine Gottschling, Andreas Faissner

**Affiliations:** ^1^Department of Cell Morphology and Molecular Neurobiology, Faculty of Biology and Biotechnology, Ruhr-University, Universitätsstraße 150, 44801 Bochum, Germany; ^2^International Graduate School of Neuroscience, Building FNO 01/114, Ruhr-University, Universitätsstraße 150, 44801 Bochum, Germany

## Abstract

Synapses are specialized structures that mediate rapid and efficient signal transmission between neurons and are surrounded by glial cells. Astrocytes develop an intimate association with synapses in the central nervous system (CNS) and contribute to the regulation of ion and neurotransmitter concentrations. Together with neurons, they shape intercellular space to provide a stable milieu for neuronal activity. Extracellular matrix (ECM) components are synthesized by both neurons and astrocytes and play an important role in the formation, maintenance, and function of synapses in the CNS. The components of the ECM have been detected near glial processes, which abut onto the CNS synaptic unit, where they are part of the specialized macromolecular assemblies, termed perineuronal nets (PNNs). PNNs have originally been discovered by Golgi and represent a molecular scaffold deposited in the interface between the astrocyte and subsets of neurons in the vicinity of the synapse. Recent reports strongly suggest that PNNs are tightly involved in the regulation of synaptic plasticity. Moreover, several studies have implicated PNNs and the neural ECM in neuropsychiatric diseases. Here, we highlight current concepts relating to neural ECM and PNNs and describe an* in vitro *approach that allows for the investigation of ECM functions for synaptogenesis.

## 1. Introduction

The development of the mammalian brain unfolds in a sequence of precisely orchestrated steps. After neurogenesis and gliogenesis neurons have migrated to their final destinations. Thereafter, the process of axon growth and guidance generates a complex network of connections that is crucial for correct central nervous system (CNS) function. Axons terminate in synapses that mediate the transfer and the storage of information. The synapse thus represents the central functional element of the nervous system. It consists of a presynapse, the synaptic cleft, and the postsynaptic membrane [1–3]. This functional unit is formed during development of the nervous system and is subject to malleability in the adult nervous system [3, 4]. There, modifications of synaptic connections on the functional and the structural level are believed to underlie synaptic plasticity that plays a key role in the context of learning and memory [3]. A whole range of cell adhesion molecules are involved in synapse formation and maturation [5]. Synaptic machinery is an intricately organized mechanism where transmembrane proteins work in concert with pre- and postsynaptic factors including cytokines [2, 6], Eph-kinases and ephrin ligands [7], cell adhesion molecules [8–11], neurexins and neuroligins [12], extracellular matrix (ECM) glycoproteins [13, 14], complementary integrin receptors [15], Narp/NP2, wnt7A, and FGF22 [3, 16], and several intracellular scaffolding molecules that anchor postsynaptic receptors [1, 17, 18]. The role of guidance molecules in synapse formation and plasticity was previously reviewed [19], and in this paper we will focus on the role of ECM in neural plasticity.

ECM provides a highly organized environment that mediates a broad spectrum of intercellular interaction in the CNS. In a subpopulation of neurons ECM develops into a specific formation termed perineuronal nets [20, 21]. Perineuronal nets (PNNs) were described by Camillo Golgi as a honeycomb-like precipitate around a subpopulation of silver-stained neurons [22]. The latter includes fast spiking GABAergic interneurons expressing parvalbumin [23, 24], and sometimes other types of neurons, for example, excitatory pyramidal neurons, can exhibit these macromolecular structures [25, 26]. PNNs localize to the soma and dendrites and delineate synapses on neuronal surfaces, which led to the hypothesis that PNNs contribute to the regulation of neuronal plasticity [22, 27]. Their function is based on the net-like assembly of ECM components that are heterogeneously expressed [28, 29] and interact with the pericellular microenvironment and the surrounding cells. Both neurons and astrocytes contribute to the formation of the tripartite synapse [30]. In the past years, numerous studies have been published examining the structure and function of PNNs in the central nervous system. Within the present review, we will focus on our approach to study the neural ECM in PNNs in the context of astrocyte-neuron interactions and their regulatory function in the establishment of synaptic connections and their maintenance and plasticity.

## 2. ECM of the CNS

The ECM is composed of glycoproteins and proteoglycans that form an interactive network of macromolecules for which the term matrisome has been proposed [20, 21]. According to this description, the matrisome core comprises about 300 genes whose products structure the extracellular space and function as a scaffold for the binding of various molecular ligands and cells. While it had originally been thought that the ECM of the CNS is confined to the basal lamina of blood vessels and the meninges, a wealth of data has meanwhile shown that it plays crucial roles in the neural stem cell compartment [31, 32], in axon growth and guidance [33], in the visual system [34], and in the lesion response of the CNS [35–37]. The ECM of the CNS consists of glycoproteins including laminins, tenascins, thrombospondins [33, 38], and proteoglycans. The latter ones comprise a core protein and at least one covalently linked unbranched glycosaminoglycan (GAG) chain, which defines the subtypes of heparan sulfate proteoglycans (HSPGs) [39–42] and chondroitin sulfate proteoglycans (CSPGs) [43–45]. In particular CSPGs of the lectican family such as aggrecan [46–54], brevican [55–59], neurocan [46, 55, 60–62], and versican [46, 55, 61, 63, 64] (for a detailed review concerning lecticans see [65]) are abundantly expressed in the developing CNS and enriched in (PNN) structures [66]. These are thought to be involved in processes such as ion-buffering [67], connection to the intracellular cytoskeleton [22], protection against oxidative stress [68], and stabilization of synapses [69] (see below). With the exception of one splice variant of brevican that is anchored to the plasma membrane via GPI [59], all members of the lectican family are secreted into the extracellular space [45].

## 3. PNNs Composition and Structure

**Figure 1:**
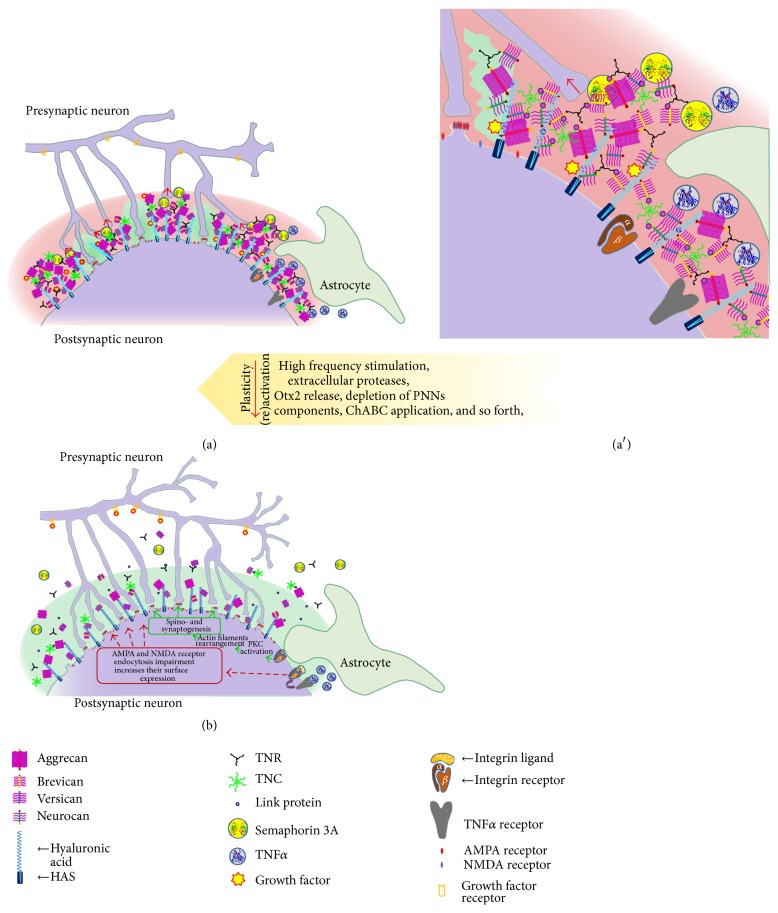
PNNs and neural plasticity. The cartoon depicts the composition of an ECM coat in a PNN on neuronal surfaces, as produced jointly by neurons and astrocytes (a), magnified in (a′). PNNs restrict adult neuronal plasticity, by providing inhibitory environment (depicted in red) restricting astrocyte-induced plasticity and by embedding repulsive guidance molecules. Only several permissive areas are left, indicated in green. Remodelling of PNNs and consequent regain of plasticity (b) can be induced by distinct treatments, as shown by the arrow.

In close proximity to certain types of CNS neurons, the diffuse distribution of ECM changes towards a highly condensed configuration, creating a specific formation termed PNNs [20, 21]. These assemblies are identified as the areas of dense immunocytochemical staining for one of their core molecular components. The most widely used markers to detect PNNs include* Wisteria floribunda* agglutinin (WFA) lectin and antibodies against CSPG core proteins [24, 70, 71]. Although the expression of PNNs components displays some heterogeneity throughout different brain regions [29, 72], several of them can be defined as core components [66, 73]. In particular, the CSPGs of the lectican family [28] are highly enriched in PNNs and share a conserved globular domain at their N-terminus whereby they interact with hyaluronic acid (HA) [49, 55, 65, 74–76], another core component of PNNs (Figure 1). The HA is composed of disaccharides consisting of N-acetylglucosamine and glucuronic acid that forms a linear structured polymer [36, 77] which is not bound to a core protein [36, 78–80]. According to recent reports the membrane based hyaluronic acid synthase (HAS) is at least in part responsible for the attachment of PNNs to the neuronal membrane via binding interactions of PNN constituents such as the lecticans with HA [36, 74, 81]. A further constituent of the PNNs is the CSPG termed DSD-1-PG/phosphacan [55, 66, 82–86]. Phosphacan is a splice variant of the receptor protein tyrosine phosphatase- (RPTP-) *β*/*ζ*, a transmembrane receptor linked to several relevant signal transduction pathways [43, 87].

Phosphacan interacts with other ECM constituents, namely, the tenascin glycoproteins, which are also compounds of PNNs. The glycoprotein tenascin-R (Tnr) of the tenascin gene family [48–50, 55, 88–92] is a further prominent component within PNN structures. Tnr has so far exclusively been detected in the CNS where it occurs as a trimeric glycoprotein [90, 92]. Tnr displays binding sites for members of the lectican family, for example, versican [93], brevican [94], and neurocan [95], and has the potential to cross-link ECM components due to its trimeric structure [13].

Link proteins are important for enhancing and maintaining the interactions of CSPGs with HA and involved in the formation of PNNs because thereby they increase the stability of the PNN structure [55, 74] (Figure 1). HAPLN1 (HA and proteoglycan link protein 1)/Crtl1 (cartilage link protein 1), HAPLN2/Bral1 [96], and HAPLN4/Bral2 (brain link proteins 1 and 2) [97] are the most thoroughly studied link proteins relating to PNNs and are known for their interaction with CSPGs and HA [48, 55, 98–101]. Summarizing these indications, it can be stated that the CSPGs of the lectican family, as well as HA, Tnr, and link proteins, determine the scaffold of PNNs in the central nervous system by establishing intensely structured extracellular aggregates [28, 55, 102].

Importantly, a number of regulatory molecules are associated with PNNs. These variable elements can be linked to the main components by either direct interaction with core protein or by binding with GAG chains. One example is tenascin-C (Tnc) that is expressed during the development of the central nervous system [86, 103–108]. Also the glycoprotein semaphorin 3A [76, 109–111] (see below), which plays a crucial role in the process of axon guidance [112, 113], is attached to molecules of PNNs, in particular proteoglycans [114]. Other PNN-associated molecules, in particular matrix metalloproteases (MMPs) and Otx2, also contribute to neural plasticity and will be discussed further in this review.

## 4. Astrocyte-ECM-Neuron Interactions in Neuronal Plasticity

The impact of astrocytes on neuronal networks development, their regulation, and plasticity has been a subject of intensive research throughout the last decades [115]. As the new insights were provided, our understanding of glia has switched from an intercellular “glue” to an active component of the CNS [30, 116–118]. We now know that astrocytes not only provide neuronal networks with essential structural and metabolic support, but also modulate neuronal activity and neural plasticity [30, 69, 119–121] and support the formation of neuronal circuits [111, 122]. In addition, astrocyte-neuron crosstalk is crucial for neuroprotection and is involved in neurological diseases progression in several cases [119, 120]. According to the current concept of the tripartite synapse astrocytes are necessary for the establishment and regulation of synapses [69, 117, 122–125]. Furthermore, glial subpopulations can also directly form synapses with neurons [126]. Neurons contact astrocytes in multiple ways including ephrin-based interactions [127] and ECM-mediated integrin signalling [128]. ECM molecules mediate a substantial part of astrocyte-neuron interactions and to consider them as independent entity in a conceptual construct that has been termed the tetrapartite synapse has been proposed [69, 119, 123].

**Figure 2:**
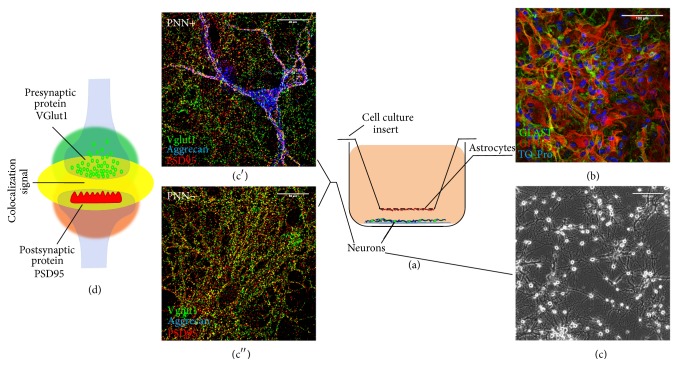
Neuron-astrocyte coculture for the study of synaptogenesis. A schematic view of the neuron-astrocyte indirect coculture system is presented. (a) Primary embryonic day 15 mouse hippocampal neurons are cultivated on coverslips in the presence of primary cortical astrocytes maintained as monolayers in cell culture inserts (b). Thereby, astrocytes and neurons share the same medium in the absence of membrane-mediated contacts. With the use of this system, neurons can be cultivated for up to 4 weeks and form active neuronal networks (c) in completely defined media [132], suggesting a reliable model for synaptogenesis studies. A subgroup of neurons can develop PNNs, as indicated by a specific marker (c′ and c′′). Presynaptic and postsynaptic terminals can be visualised using immunocytochemical labelling of presynaptic and postsynaptic proteins. The overlap of pre- and postsynaptic puncta indicates the structural synapses (d). Quantification of synaptic puncta using an analysis software permits the quantitative evaluation of synapse formation* in vitro* under different treatment conditions. For experimental details see [129, 130].

Indirect neuron-astrocyte coculture* in vitro* models provided a powerful tool to study astrocyte-neuronal interactions. Applying this approach, Pyka et al. [129, 130] combined pure cultures of primary embryonic hippocampus neurons with pure cultures of primary astrocytes, using cell culture inserts to avoid direct membrane-mediated cell contacts. Under these conditions neurons survived and developed neuronal networks when sharing the defined culture medium with primary astrocytes (Figure 2). The latter also supported synaptogenesis in the neuronal culture [129]. The use of this coculture system opens the possibility for numerous forms of analysis exclusively for neurons, for example, isolation of mRNA for qRT-PCR, analysis of the transcriptome, expression analysis of distinct proteins via Western Blotting, and immunocytochemistry, while the cells dispose of a long* in vitro* life span. Furthermore, the unique model allows the investigation of the source of ECM within the shared culture medium. Geissler et al. used an analogous model [131, 132] to reveal the impact of several ECM components on the establishment of neuronal networks and the expression of PNNs [131]. In that approach, neurons and astrocytes were obtained from either wild type or quadruple knockout mice, which lack Tnc, Tnr, brevican, and neurocan [133]. Excitatory synapse formation, postsynaptic currents, and PNNs expression were then analysed in different combinations of wild type and knockout cells. Depletion of the four crucial ECM molecules led to an impairment of PNN formation, decreased frequency of miniature inhibitory and excitatory postsynaptic currents, and disturbed synaptogenesis. Indeed, an initial increase of synapse formation after two weeks was followed by the marked decrease of synapse numbers, an effect that was particularly prominent with PNN-bearing neurons, indicating that the latter may be important for synapse stabilization in the long run [131].

Interestingly, the effects of the quadruple knockout were prominent in knockout neurons and could not be rescued by sharing the secretome of wild type astrocytes [131]. As four genes are depleted in the quadruple knockout mouse, it is difficult to attribute the phenotypic changes to one of the missing ECM constituents. Thus, the knockout of neurocan [133] causes only subtle modifications regarding the late phase in long-term potentiation maintenance, without affecting PNNs or brain development [134]. Moreover, the triple knockout of Tnc, Tnr, and versican generates a similar phenotype as the quadruple knockout [133]. Interestingly, neurocan was almost absent in triple knockout mouse brains. Fibulin-1 and fibulin-2 are upregulated and localized interstitially in the quadruple knockout brain, which may exert a compensatory effect by transiting from a tenascin- to a fibulin-cross-linked ECM [133].

## 5. PNNs in Neural Plasticity

A number of regulatory functions of the ECM that is collectively synthesized by neurons and glia are attributed to PNNs (Figure 1). Interestingly, these macromolecular assemblies can modulate different types of neuronal plasticity, including circuit remodelling and synaptic plasticity [73, 80, 135]. During CNS development, neuronal plasticity is required for controlled remodelling of neuronal circuits. At this level, the role of PNNs was abundantly demonstrated* in vivo* in the context of ocular dominance plasticity (for review see [73]). It has been noted that the expression of molecules forming the PNNs coincides with the closure of the critical period during the development of the brain [73, 80, 135–138]. Increasing evidence also suggests that PNNs are remodelled in correlation with activity [139, 140]. On the structural level, there is evidence that PNNs restrict neurite growth and the development of synapses [141, 142]. On the synapse level, PNNs compartmentalize the neuronal surface and restrict glutamate receptor mobility [81, 143, 144], thus providing support for synaptic plasticity and stabilizing synapses [37, 142]. Preventing the mobility of AMPA receptors led to a reduction of short-term plasticity in rat primary neurons [80, 143, 145], indicating the possible role of PNNs for memory formation. A number of PNN components were shown to regulate synaptic plasticity in their own right. Neurocan deficiency reduced the stability of late-phase LTP [134], brevican ablation led to significantly impaired LTP [146], and depletion of its binding partner Tnr also caused a reduced LTP [147]. Interestingly, knockout of the related glycoprotein Tnc led to a complete failure in LTD induction, together with impaired LTP development [148]. It was hypothesized that the consequence of the Tnc-knockout was due to the reduced L-type VDCC channel signalling.

### 5.1. Enzymatic Digestion of PNNs Induces Neural Plasticity

The bacterially derived enzyme chondroitinase ABC (ChABC) has been used in numerous studies for analysing the role of PNNs in neuronal plasticity [138, 149]. This enzyme degrades especially the GAG chains of CSPGs, more precisely chondroitin-6-sulfate, chondroitin-4-sulfate, dermatan sulfate, and HA [150], depending on the pH optimum of the enzyme (pH 8.0: chondroitin sulfate; pH 6.8: HA) [151] without altering the core proteins of the ECM. This degradation results in enhanced neuronal plasticity, for example, a higher expression of synaptic proteins in rat hippocampal neurons [130], restoration of ocular dominance plasticity in the adult cat visual cortex [136, 152], and enhanced regeneration of sensory projections and corticospinal tract axons within the adult rat spinal cord after lesion [37, 153]. The inhibitory effects of CSPGs on synapse formation and plasticity could be caused by the chondroitin sulfate GAG (CS-GAG) chains with varying degree of epimerization and sulfation that might result in functional subdomains along the polymer. These domains could interact with and thereby expose a wide range of protein ligands, including the inhibitory semaphorins (see below) [154, 155]. Alternatively, they can also directly activate specific receptors (for review see [156]) that mediate growth cone stalling or retraction, for example, RPTP*σ* [157]. In spinal cord lesions, the modulation of RPTP*σ* promotes recovery after spinal cord injury [158].

### 5.2. PNNs Restrict Integrin Signalling

Apart from the compartmentalization of the neuronal surface PNNs also control synaptogenesis through integrin signalling. Astrocytes can make contacts with neurons via integrin receptors. Focal integrin activation further leads to global PKC activation, resulting in excitatory synaptogenesis facilitation [21, 128, 159]. Interestingly, CSPGs that are abundantly expressed in PNNs inhibit integrin activation [36, 47]. Moreover, there is an evidence for direct interaction between CS-GAGs and integrins, for example, the interaction of the integrin *α*_4_ *β*_1_ and the melanoma chondroitin sulfate proteoglycan [160, 161]. A number of alternative ways of how CSPGs may reduce integrin activation have been suggested [162]. From this point of view, modulating the CSPG coating provides another ECM-mediated mechanism of astrocyte-dependent control over neuronal synaptogenesis [69, 130].

### 5.3. PNNs-Associated Molecules and Neural Plasticity

Beyond integrin signalling, PNNs can mediate molecular signals between neurons and astrocytes via a number of regulatory molecules that they capture. A number of growth modulating ligands interact with CS-GAGs, including PTN, FGF, and EGF isoforms [43, 154, 155, 163]. Further ligands include TNF*α* [164], BDNF, semaphorins [165], and extracellular matrix proteases [164]. Semaphorins are particularly interesting because members of this gene family, notably semaphorin 3A which is synthesized by both neurons and astrocytes, are growth cone collapse inducing molecules with a strong impact on axon growth and guidance [109, 114, 166], axon regeneration [110], establishment of neuronal polarity [112], and the development of dendritic spines [167]. Semaphorin 3A can synergize with CSPGs to regulate neuronal migration [168] and abounds in PNNs [114, 165, 169]. There, it binds to chondroitin sulfate E GAGs (CSE-GAGs), a disulfated disaccharide epitope (GlcUA-GalNAc(4S,6S)), also named E unit [169–171], and could contribute to the repulsion of synaptic sprouts and inhibition of synaptogenesis [37, 113]. Release of semaphorin 3A by ChABC could reduce the inhibitory properties of PNNs and thus may explain increased synaptogenesis upon ChABC treatment [69, 130].

Along these lines, several of the discovered CSPG-ligands are known to modulate synaptic plasticity and are released from astrocytes. For example, TNF*α* is involved in astrocyte-mediated synaptic scaling [172]. In response to the absence of presynaptic potential astrocytes release TNF*α*, which leads to elevated expression of *β*3-integrins at the postsynaptic site. Integrins further enhance AMPARs surface expression through RAP1 inhibition, which leads to the upscaling of postsynaptic currents [6, 172, 173]. ECM proteases, including MMPs, ADAMTS, and TPA, often show a similar mode of action, although their substrates differ. They exhibit elevated expression after LTP induction and support memory formation and learning. Extracellular proteases are not mainly responsible for ECM degradation but rather regulate neural plasticity through cleaving signalling motifs and active forms of growth factors. The reader is welcome to address a recent review for further information [174]. Remarkably, the metallopeptidases Adamts8, Adamts15, and neprilysin are expressed in fast spiking parvalbumin interneurons that are surrounded by PNNs. It is an intriguing possibility that these proteases may partake in PNN remodelling, in dependence of neuronal activity affecting their release [175].

Another intriguing PNN-associated molecule that regulates neural plasticity is Otx2 [176, 177]. This homeobox-containing protein is involved in transient reopening of the visual plasticity period in amblyopic mice [178]. When an arginine-lysine doublet peptide is infused, Otx2-localization to PV-containing interneurons is disrupted, PNNs expression is decreased, and neuronal plasticity is reopened in adult mice, comparable to the situation after ChABC treatment [176, 177]. These results clearly speak in favour of a control of plasticity by interneuronal Otx2 transfer [179–181].

### 5.4. PNNs and Ionic Homeostasis

The polyanionic nature of PNNs introduces them as an important element of an neuronal excitability regulatory system [67, 68]. While astrocytes actively remove K^+^ and neurotransmitters from the extracellular space, preventing overexcitation [119, 182], PNNs buffer cations in close proximity to neuronal membrane, thus enabling rapid spiking [139, 183, 184]. Together with the fact that PNNs are often found around fast depolarizing Kv3.1b channel expressing interneurons, this observation clearly indicates the potential of this macromolecular buffer for the regulation of inhibition by the interneurons. Moreover, being highly hydrated polyanions some compounds of PNNs are needed to maintain brain extracellular space [67]. In particular, reduction of hyaluronic acid synthesis upon HAS knockout led to severe brain extracellular space reduction, diffusion impairment, and epileptiform activity [185].

To summarize, astrocyte-ECM-neuron interaction provide, to our current knowledge, four main groups of mechanisms to modulate neural plasticity: (i) compartmentalization of neuronal surface to restrict and to stabilize synapse formation; (ii) synaptogenesis restriction through integrin signalling suppression; (iii) mediation of molecular guidance signals for synaptogenesis and synaptic plasticity; (iv) ionic gradients and extracellular space maintenance. All these mechanisms are crucial for CNS development, function, and homeostasis. It is no surprise that malfunctioning of this three-party orchestra can cause multiple neurological diseases, which will be addressed in a later paragraph (see below).

## 6. Cellular Origins of PNNs Components

The assumed functions of PNNs raise the question about which cellular compartments contribute to their construction. In the realm of the tripartite synapse both astrocytes and neurons could be the source of secreted ECM building blocks of the PNNs (Figure 2). This poses questions relating to their relative roles and the regulatory mechanisms involved in these distinct cellular compartments. To understand how the interplay between glia, neurons, and PNNs affects neuronal plasticity, it is necessary to identify the cellular sources of PNNs components. So far, only few researchers addressed this question [49, 51, 55]. For this reason, we will review the available data about the expression of PNNs components by astrocytes and neurons under physiological conditions (Table 1).

Miyata et al. first showed [51] that cultured neurons can express PNNs in the absence of astrocytes. Using pure cultures of rat cortical neurons, they found the presence of WFA lectin labelled PNNs around parvalbumin-containing interneurons. These PNNs also contained neurocan, phosphacan [85, 87], neuroglycan C, and HA. The expression of PNNs increased as the neurons maturated, matching the timelines of their development* in vivo*. Importantly, under these experimental conditions, WFA labelling surrounded synapsin puncta in immunofluorescence stained preparations. This evidence indicates that the development of structurally integral PNNs does not depend on astrocytes.

Further, Carulli et al. [55] addressed the question of cellular sources of PNN components in rat cerebellum. The authors combined immunohistochemistry with* in situ* hybridization to determine which cell types express mRNAs encoding a variety of proteins contributing to PNNs formation. Briefly, neurocan, aggrecan, and link proteins Crtl1 and Bral2 were expressed by neurons, not glial cells. Versican and phosphacan mRNA localized to NG2 glia and oligodendrocytes but was absent in neurons. Brevican mRNA was found in neurons and astrocytes, while Tnr was expressed by NG2 glia, oligodendrocytes, and neurons. It is interesting to note that link proteins Crtl1 and Bral2, originating from neurons, are crucial for PNNs structural integrity [74, 186]. In the absence of one of these two proteins, PNNs are attenuated and neuronal plasticity is enhanced [98, 187].

Addressing this issue, Giamanco and Matthews [49] have carefully addressed the question of cellular sources of PNNs components. Applying AraC and KCl in different combinations to mixed cultures of mouse cortical glia and neurons, the authors dissected neuronal and glial contributions to PNNs formation. Briefly, Tnr, neurocan, versican, phosphacan, brevican, Crtl1, Bral2, and HAPLN3 appeared to be expressed in a glia-dependent manner, whereas aggrecan expression was neuron-dependent. Interestingly, HA-synthesis was both neuron- and glia-dependent. However, these data do not rule out that glia-dependent components can be produced by neurons. Although only a limited number of articles focused on the study of cellular origins of PNNs components, accessory information can be drawn from a number of other studies. A thorough search in literature databases targeting the expression of major PNNs compounds in different types of cells of the mammalian CNS under physiological conditions provided further insight. We have further verified whether the cellular origin of a component of our interest was clearly stated. The results of our search are summarized in Table 1.

Summarizing the available reports, both neurons and glial cells can produce the majority of PNNs components, while neurocan and link proteins Crtl1 and Bral2 seem to be neuron-specific, at least* in vivo* and under physiological conditions. It is important to note, however, that the expression profile of PNNs components may change upon activation of glia or under conditions of neuronal hyperactivity. For instance, when glial activation occurs upon brain injury or experimental stress conditions, astrocytes start to express sustainable levels of neurocan [61, 188–190], and phosphacan expression is highly upregulated in reactive astrocytes [82, 189, 191, 192].

## 7. Neurologic Diseases and PNN Formation

Several neurologic diseases have been identified to exhibit an impaired PNN formation* in vitro* and* in vivo,* including epilepsy and schizophrenia (for detailed review see [145]). As a consequence of the fact that PNN formation and maintenance underlie alterations in these diseases, neuronal plasticity is also affected, which, for example, is the case in epilepsy. Here, the disease is accompanied by epileptic seizures triggered by an altered GABAergic signalling [193] together with an abnormal expression and functionality of GABA receptor seen in primary dentate granule cells in a temporal lobe epilepsy rat model [194–196]. A compromised signalling capacity of GABAergic neurons could also be found in a rat model of Alzheimer's disease, in which rats were treated with amyloid beta [197]. Moreover, animal models regarding the psychiatric disease schizophrenia demonstrate an alteration of the inhibition of GABA together with a loss of parvalbumin-expressing neurons [198–200]. As emphasized previously, PNNs enwrap GABAergic neurons [24, 26, 67].

In addition, several PNN components display a modified expression in animal models mimicking epilepsy and schizophrenia, which is consistent with a change of neuronal plasticity. Different expression patterns of neurocan and phosphacan were observed in the hippocampus in a temporal lobe epileptic rat model compared to healthy control rats [201]. Another study also showed an impairment of neurocan and phosphacan, as well as Tnc, using a different mouse model of epilepsy, in which domoate, a specific glutamate agonist, is injected next to the hippocampus* in vivo* [202]. Here, immunohistochemical staining revealed a significantly higher expression of neurocan and Tnc seven days after injection, whereas the expression of phosphacan was not increased until 14 days after the injection [202]. Reports in the literature suggest that neurocan expression within hippocampal regions is increased in parallel with a decreased phosphacan expression, followed by the death of pyramidal cells after kainic acid application in rats [203]. PNN formation during schizophrenia is altered in that PNN densities, as revealed by immunohistochemical staining of human postmortem brains with WFA, are highly diminished in the prefrontal cortex of schizophrenic patients [204]. The importance of the existence of a healthy composition of PNNs is shown in another study analysing human postmortem brains of patients suffering from schizophrenia by immunohistochemistry. There, the authors found a higher number of glial cells positive for CSPGs in the amygdala and the entorhinal cortex, but also a decreased density of PNNs in parts of the amygdala and the entorhinal cortex [72]. A continuative study of this group could recently examine a reduction of glial cells positive for aggrecan in the schizophrenic amygdala [205].

These findings contrast the composition of PNNs in the brain of Alzheimer's disease patients. Recently, a study uncovered that PNNs in postmortem brains were unaffected, in particular with regard to the expression of brevican, aggrecan, Tnr, and Crtl1, whereas HA displayed an enhanced expression in amyloid beta plaque areas [206]. In light of these findings, the authors suggested that PNNs might exert a neuroprotective function for the neurons of Alzheimer's disease patients. In future, a substantial effort will be required to unravel the relations between altered PNN formation and neurological diseases in order to gain insight into useful therapeutic strategies.

## Figures and Tables

**Table 1:** Cellular sources of the major PNNs components.

Name	Neurons		Astrocytes		Oligodendrocytes		NG2 glia		Citations
	*In vitro*	*In vivo*	*In vitro*	*In vivo*	*In vitro*	*In vivo*	*In vitro*	*In vivo*	
HA	++	++	++	++	−	−	−	−	[49, 55, 74–76]
Aggrecan	++	++	++	−	−	−	−	−	[46–51]
Brevican	−	+^1^	+++	+++	−	−	−	−	[55–57]
Neurocan	+++	+++	+^2^	−	−	−	−	−	[46, 55, 60, 61]
Phosphacan	−	++	−	++	−	++	−	++	[55, 82, 83]
Versican	+^3^	−	+^3^	−	+++	+++	−	++	[46, 55, 63]
Tnr	++	++	+^4^	−	++	++	−	++	[48–50, 55, 88, 89]
Tnc	−	+^5^	+++	+++	−	−	−	−	[103–106]
Crtl1	−	+++	−	−	−	−	−	−	[48, 55, 98]
Bral2	−	+++	−	−	−	−	−	−	[48, 55, 98]
Semaphorin 3A	−	++	++	++	−	−	−	−	[76, 109–111]

Symbol +++ indicates the evidence for strong protein and/or mRNA expression, almost restricted to a certain cell type; symbol ++ indicates moderate expression under physiological conditions; + indicates weak or transient expression in a particular cellular subtype or under certain experimental conditions, indicated by superscript footnotes and described below. Dashes indicate the absence of evidence for cell type specific expression published so far.

^1^Neurons of molecular layer of cerebellar cortex and by large excitatory deep cerebellar nuclei neurons [55].

^2^Astrocytic monolayers in culture [61].

^3^Neurons and astrocytes differentiated from embryonic stem cells [46].

^4^Type 2 but not type 1 astrocytes [89].

^5^Transient expression by neurons of spinal cord and hippocampus during development [103, 105].
